# Clinical efficacy of laparoscopic closed hernia ring combined with a patch repair for Gilbert type III indirect inguinal hernia

**DOI:** 10.3389/fsurg.2023.1190788

**Published:** 2023-05-15

**Authors:** Luo Binyu, Zhang Qin, Zhang Xiao, Zhang Daquan, Guo Qing, Yu Jing, Tian Yunhong, Ren Mingyang

**Affiliations:** ^1^ Department of Gastrointestinal Surgery, Affiliated Nanchong Central Hospital and The Second Clinical Medical College of North Sichuan Medical College, Nanchong, China; ^2^ Department of Rehabilitation Medicine, Affiliated Hospital of North Sichuan Medical College, Nanchong, China

**Keywords:** laparoscopy, Gilbert type III indirect inguinal hernia, closed hernia ring, seroma, postoperative pain

## Abstract

**Purpose:**

The incidence of seroma and postoperative pain after Gilbert type III inguinal hernia repair is high. To reduce postoperative complications, this study investigated the clinical efficacy of laparoscopic closed hernia ring combined with a patch repair for Gilbert type III indirect inguinal hernia.

**Methods:**

Through a prospective randomized controlled study, a total of 193 patients with Gilbert type III indirect inguinal hernia admitted to Nanchong Central Hospital affiliated with Chuanbei Medical College from May 2020 to December 2021 were selected and randomly divided into the inner ring closed group (85 patients) and the inner ring non-closed group (95 patients). The patients in both groups underwent laparoscopic tension-free repair of their inguinal hernias. General information such as operative time, postoperative hospital stay, and hospital cost were compared between the two groups, and the patients were followed up at 1, 7, 14, 21, and 28 days and then 3, 6, and 12 months after surgery to compare complications such as incidence of seroma, volume of the seroma fluid, incidence of pain, and visual analogue scale (VAS) pain score.

**Results:**

There was no conversion to open procedures in any of the patients. The operation time of the closed group was significantly longer than that of the non-closed group (64.2 ± 12.2 vs. 55.3 ± 9.5 min, *P* < 0.01). The proportion of patients with postoperative pain in the two groups was 39 (46%) vs. 59 (62%), *P* = 0.029 on 7 days; 17 (20%) vs. 33 (35%), *P* = 0.028 on 14 days; and 6 (7%) vs. 22 (23%), *P* = 0.003 on 21 days in the postoperative closed group and was significantly lower than that in the non-closed group, while we found that the non-closed group had a higher VAS pain score than that of the closed group (2.36 ± 0.61 vs. 1.95 ± 0.71, *P* = 0.003 on 7 days and 2.12 ± 0.49 vs. 1.65 ± 0.49, *P* = 0.002 on 14 days) after surgery according to the statistical results of the VAS pain score. The incidence of postoperative seroma and the amount of seroma fluid decreased gradually in both groups, but when comparing the two groups, the proportion of cases of seroma in the closed group on 7 days [45 (53%) vs. 79 (83%), *P* < 0.01]; 14 days [23 (27%) vs. 43 (45%), *P* = 0.011]; and 21 days [10 (12%) vs. 29 (31%), *P* = 0.002] after the operation were significantly less than that in the non-closed group. For the comparison of the amount of seroma fluid between the groups, the seroma fluid volume in the non-closed group was greater than that in the closed group (34.48 ± 20.40 vs. 43.87 ± 16.40 ml, *P* = 0.006, 7 days) and (21.79 ± 8.42 vs. 30.74 ± 10.39 ml, *P* = 0.002, 14 days) after surgery. There were no differences in the length of stay, total hospital costs, or postoperative complications (urinary retention, intestinal obstruction, nausea, vomiting, bleeding, and infection) between the two groups, and the differences were not statistically significant (*P* > 0.05). The postoperative follow-up period was 3–20 months, and no chronic pain or recurrence occurred during the postoperative follow-up period in either group.

**Conclusions:**

Closure of the hernia ring is safe and effective for laparoscopic hernia repair for Gilbert type III inguinal hernia, and it significantly reduces the incidence of postoperative seroma and further reduces the postoperative pain without increasing the risk of postoperative infection and recurrence.

Inguinal hernia is a common surgical disease in clinical settings. Every year, there are more than 20 million cases of inguinal hernia repair worldwide (1). With the rapid development of laparoscopic minimally invasive technology in the field of hernia surgery, including endoscopic total extraperitoneal patch plasty (TEP) and laparoscopic transabdominal patch plasty (TAPP) techniques, it has become one of the gold standards for inguinal hernia repair. Currently, laparoscopic inguinal hernia repair (LIHR) has become one of the mainstream surgical methods for inguinal hernia due to its advantages of quick postoperative recovery, low rate of postoperative infection, short hospital stay, mild postoperative pain, low recurrence rate, and good curative effect (2, 3). However, for the cases of large defects of the hernia ring, such as medial defects, large indirect hernias, or scrotal hernias, complications such as seroma formation, pain, infection, and recurrence after laparoscopic repair cannot be ignored. The main postoperative complication was the development of seroma, with an incidence of 3.7%–70%. Although most seromas are considered to be common and minor postoperative complications, a large number of seromas may cause postoperative pain, infection, and even recurrence and reduce the quality of life of patients after surgery (4, 5).

Therefore, it is of great significance to identify the factors related to seroma formation. For direct hernias, there is a clear evidence to support the findings that when the lax transverse fascia is inverted, the incidence of seroma is significantly reduced when the dead space volume is reduced by a direct suture closure of the defect (6). However, for large indirect hernias (Gilbert type Ⅲ) or scrotal hernias, the methods of reducing seroma formation remain undefined, and some scholars have found no obvious improvement by transecting the hernia sac, completing dissection of the hernia, placing a drainage tube, applying prophylactic perforation ice compression, etc. In recent years, some studies have found that by closing the inner ring port and blocking the internal and external communication ports, the occurrence of seroma and postoperative complications can be effectively reduced (7–9), The occurrence of seroma can be effectively controlled and reduced, which will further reduce the incidence of other complications.

Therefore, the aim of this study was to prospectively assess a new simple method to evaluate the clinical effect of laparoscopic closure of the internal ring of Gilbert III indirect inguinal hernia and explore the clinical application value of this technology. This study can provide some reference for surgeons in the treatment of hernia sacs during laparoscopic hernia repair in the future.

## Materials and methods

The clinical data of 180 adult male patients with indirect inguinal hernia admitted to Nanchong Central Hospital affiliated to North Sichuan Medical College from May 2020 to May 2022 were selected by a prospective randomized controlled study. The inclusion criteria were as follows: adult male aged ≥18 years old and with unilateral indirect inguinal hernia according to preoperative physical examination and imaging examination. The inclusion criteria were as follows: primary indirect inguinal hernia classified as Gilbert III (defect size diameter ≥3 cm, including scrotal hernias); the American Society of Anesthesiologists (ASA) I, II, or III compensated; when the pneumoperitoneum induced under general anesthesia was tolerated; and when there was no obvious surgical contraindication in the preoperative examination. For the exclusion criteria, patients who could not tolerate general anesthesia, contraindications to laparoscopic surgery, direct inguinal hernia, femoral hernia, recurrent hernia, incarcerated hernia, strangulated hernia, and other hernia types were excluded; patients transferred to laparotomy were excluded; and patients who had emergency surgery were excluded. This study was reviewed and approved by the medical ethics committee of our hospital [no: 2021 annual review (016)]. All patients provided their written informed consent to participate in this study. China Clinical Trial Registration Center (chictr210049027).

## Operative technique

The same surgeon performed all the procedures. All patients were operated on under general anesthesia. The procedure for laparoscopic inguinal hernia surgery was performed in strict accordance with the guidelines for laparoscopic (TAPP) and endoscopic (TEP) treatment of inguinal hernia stipulated by the International Endohernia Society (IEHS) (10), In the selection of surgical methods for these cases, patients with ≦3 cm diameter of hernia ring defect <4 cm, no history of surgery in the lower abdomen, TEP or TAPP were selected. In addition, patients with ≦4 cm diameter of hernia ring defect, history of surgery in the lower abdomen, scrotal hernia, and irreducible hernia should be operated on with TAPP. We mainly transect the hernia sac rather than completely remove it, and only a few patients undergo complete dissection of the hernia sac. The intraoperative pneumoperitoneum pressure was set at 12 mmHg, and the pneumoperitoneum flow was set at 10 L/min. No intraoperative complications occurred during the operation.

**Figure 1–4:**
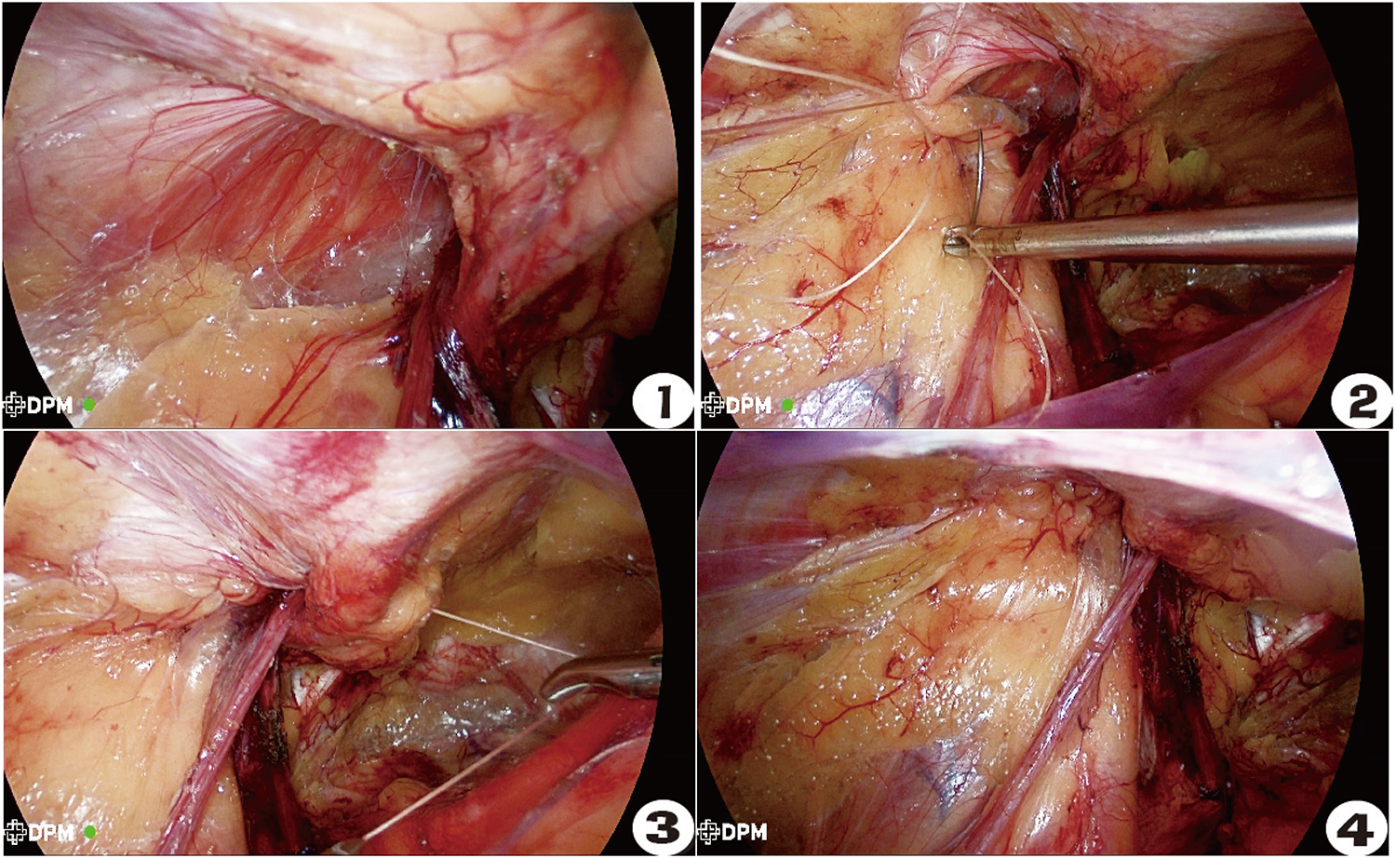
Operation procedure for closing the inner ring of Gilbert type III indirect inguinal hernia. ① Status before closing the inner ring. ② The ilio-pubic tract and conjoined tendon were sutured. ③ U-shaped suture crossing abdominal wall blood vessels. ④ The closed internal ring.

In the inner ring closure group, the pneumoperitoneum pressure was appropriately reduced, and the surgical assistant pressed the body surface to reduce the tension intensity. A 2-0 absorbable suture (V905E, VICRYL, Ethicon, USA) was used for reverse needle suturing from the lateral side to the medial side, and the combined tendon was sutured with the iliac pubic tract. The inner ring mouth was covered by the folded transverse abdominal fascia through the posterior part of the blood vessel under the abdominal wall. The suture was continuously returned to the initial section and tied for fixation. During the suturing process, the needle should be maintained at a distance that was not too far, and the needle should not be too deep in order to avoid damaging the nerve [femoral branch of the genitofemoral nerve (GFN), iliohypogastric nerve (IHN), and ilioinguinal nerve (IIN)], and the medial edge should be closed to avoid damaging the vas deferens, spermatic vessels, and inferior abdominal vessels. The spermatic cord passage should be preserved to avoid scrotal edema caused by clamping that is too tight (11). After suturing, the hernia sac was pressed externally to squeeze the gas in the distal hernia sac back into the abdominal cavity (Figures 1–4). The control group was operated on directly without closing the inner ring. We used a macroporous, partially absorbable, polypropylene mesh (15 cm × 10 cm) (PASL; TransEasy Medical Tech. Co., Ltd., Beijing, China) for the repair. No drainage tube placement and no fixation (glue or tacks) were performed in our series. The TEP surgical patch was not fixed, while the TAPP surgical patch was fixed with 3-0 VICRYL plus three-point fixation.

## Postoperative assessment

The patients were able to eat semiliquid food and get out of bed 6 h after the surgery, and the patients received regular postoperative instructions at discharge, including the use of compression dressings for 7 days, return to normal activities when able, and a temporary cessation of physical activity for 3 months.

All patients provided informed preoperative consent. Patient demographics, the diameter of the great inner ring of the hernia defect, the size of the hernia sac, the selected surgical method, such as the TAPP/TEP, the operation time, and the occurrence of intraoperative bleeding were collected prospectively. Among the available pain scores, the visual analogue scale (VAS) score is widely used. Pain scores were recorded on the first postoperative day using the VAS pain score. Outpatient follow-up visit was performed at 7, 14, 21, and 28 days after discharge, and telephone follow-up or outpatient follow-up visit was performed at 3, 6, and 12 months. The mean follow-up time for the closed group was 13.5 months, ranging from 3 to 24 months, and that for the non-closed group was 11.6 months, ranging from 3 to 22 months. The main contents of follow-up were seroma formation, seroma fluid volume {seroma size was measured by Doppler ultrasonography, and sac volume was calculated using the formula for an ellipsoid [V = (4/3) 5 abc], where a, b, and c represent the radius of the length, width, and height, respectively}, postoperative pain, VAS pain score, hernia recurrence, infection, or any serious adverse event, which were recorded and analyzed. A regular physical examination to diagnose seroma was performed at each follow-up visit by the same surgeon.

## Statistical analysis

The SPSS 25.0 software was used for analysis. Continuous variables were expressed as the mean ± standard deviation, using *t*-tests and repeated measurement data using ANOVA. Categorical variables were expressed as numbers and percentages, and the *χ*^2^ was used for the analysis of categorical variables. Fisher's exact probability method was used for small probability events. *P* < 0.05 indicates that the difference is statistically significant.

## Results

This two-year prospective study involved a total of 180 patients randomized by simple randomization into the inner ring closed group (85 patients) and the inner ring non-closed group (95 patients) (Table 1). Demographic characteristics such as age, body mass index (BMI), hernia duration, comorbidities, hernia location, and hernia size were comparable in the closed and non-closed groups (*P* > 0.05).

**Table 1:** General demographics and perioperative data of the patients.

Variable	Closed	Non-closed	*t/x2*	*P*
	(*n* = 85)	(*n* = 95)		
Age (years)	64.0 ± 14.6	65.7 ± 11.4	0.855	0.394
BMI (kg/m^2^)	24.0 ± 3.0	23.7 ± 2.9	1.000	0.319
Hernia duration (months)	62.4 ± 103.5	42.2 ± 80.9	1.469	0.144
Comorbidities				
Hypertension	13	16	0.08	0.778
Diabetes	14	17	0.064	0.801
COPD	10	14	0.343	0.558
Hernia Location			0.420	0.517
Right	72	77		
Left	13	18		
Hernia defect size (cm)	3.5 ± 0.7	3.6 ± 0.7	0.339	0.735
Length of hernia sac (cm)	9.0 ± 2.1	9.4 ± 2.0	1.311	0.191

BMI, body mass index; COPD, chronic obstructive pulmonary disease.

There was no conversion to open procedures in any of the patients. By comparing the two groups of patients, we found that the operation method (17.6% vs. 24.2%, TEP; 82.4% vs. 75.8%, TAPP), treatment of hernia sac (83.5% vs. 87.4%, transected hernia sac; 16.5% vs. 12.6%, no-transected hernia sac), blood loss (3.7 ± 4.7 vs. 3.8 ± 4.0 ml), postoperative length of stay (POLS) (2.2 ± 1.4 vs. 2.4 ± 2.0 days), and hospitalization cost (HC) (12,774.6 ± 1,009.3 vs. 12,605.0 ± 1,089.4 RMB) in the closed group were equivalent to those in the non-closed group (*P* > 0.05). However, the operation time of the closed group was significantly longer than that of the non-closed group (64.2 ± 12.2 vs. 55.3 ± 9.5 min, *P* < 0.01). The postoperative complications, including urinary retention (10.6% vs. 17.9%), intestinal obstruction (4.7% vs. 3.2%), sickness and vomiting (2.4% vs. 3.2%), incision bleeding (3.5% vs. 3.2%), and patch infection (0% vs. 0%), were comparable in the closed and non-closed groups (*P* > 0.05) (Table 2).

**Table 2:** Comparison of operative data between the two groups.

Variable	Closed	Non-closed	*t/x2*	*P*
	(*n* = 85)	(*n* = 95)		
Operation method			1.160	0.281
TEP	15 (17.6%)	23 (24.2%)		
TAPP	70 (82.4%)	72 (75.8%)		
Transected hernia sac			0.535	0.465
Yes	71 (83.5%)	83 (87.4%)		
No	14 (16.5%)	1,212.6%)		
Blood loss (ml)	3.7 ± 4.7	3.8 ± 4.0	0.042	0.967
OT (min)	64.2 ± 12.2	55.3 ± 9.5	5.405	<0.01
POLS (days)	2.2 ± 1.4	2.4 ± 2.0	1.064	0.289
HC (RMB)	12,774.6 ± 1,009.3	12,605.0 ± 1,089.4	1.080	0.282
Complications				
Urinary retention	9 (10.6%)	17 (17.9%)	0.288	0.592
Sick and vomit	2 (2.4%)	3 (3.2%)	0.108	0.743
Incision bleeding	3 (3.5%)	3 (3.2%)	0.019	0.890
Patch infection	0	0	-	-

POLS, postoperative length of stay; HC, hospitalization cost; OT, operative time.

Pain is an inevitable complication after inguinal hernia surgery. The recovery of the two groups was objectively compared by the VAS pain score. The statistical results showed that the number of postoperative pain cases and VAS scores of the two groups of patients showed a downward trend with time at 1, 7, 14, 21, and 28 days and 3 months after the operation. However, the proportion of the patients with pain at 7 days [39 (46%) vs. 59 (62%), *P* = 0.029]; 14 days [17 (20%) vs. 33 (35%), *P* = 0.028]; and 21 days [6 (7%) vs. 22 (23%), *P* = 0.003] after operation in the closed group was significantly lower than that in the non-closed group (Table 3). At the same time, we showed that the VAS pain score in the non-closed group was higher than that in the closed group (2.36 ± 0.61 vs. 1.95 ± 0.71, *P* = 0.003, 7 days) and (2.12 ± 0.49 vs. 1.65 ± 0.49, *P* = 0.002, 14 days) after surgery according to the statistical results of the VAS pain score (Table 4)**.**

**Table 3:** Comparison of cases of postoperative pain between the two groups.

Group	Number of cases of postoperative pain (%)						
	1 day	7 days	14 days	21 days	28 days	3 m	6 m
Closed (*n* = 85)	63 (74%)	39 (46%)	17 (20%)	6 (7%)	5 (6%)	1 (1%)	0
Non-closed (*n* = 95)	77 (81%)	59 (62%)	33 (35%)	22 (23%)	11 (12%)	2 (2%)	0
*t*	1.248	4.760	4.856	8.851	1.798	-	-
*P*	0.264	0.029	0.028	0.003	0.180	-	-

**Table 4:** Comparison of postoperative VAS pain scores between the two groups.

Group	VAS pain score				
	1 day	7 days	14 days	21 days	28 days
Closed (*n* = 85)	3.06 ± 1.06	1.95 ± 0.71	1.65 ± 0.49	1.20 ± 0.45	1.40 ± 0.55
Non-closed (*n* = 95)	3.08 ± 0.89	2.36 ± 0.61	2.12 ± 0.49	1.23 ± 0.43	1.09 ± 0.30
*t*	0.088	3.033	3.259	0.127	1.476
*P*	0.930	0.003	0.002	0.900	0.162

Postoperative seromas usually occur 7 days after the inguinal hernia surgery. The incidence of postoperative seroma and the amount of seroma fluid in the two groups decreased gradually within the group, but compared between the two groups, the proportion of patients with seroma in the closed group on 7 days [45 (53%) vs. 79 (83%), *P* < 0.01]; 14 days [23 (27%) vs. 43(45%), *P* = 0.011]; and 21 days [10(12%) vs. 29(31%), *P* = 0.002] after the operation were significantly less than that in the non-closed group; however, there was no difference in the proportion of patients who developed seromas after surgery between the two groups [4 (5%) vs. 10 (11%), 28 days) (*P* > 0.05] (Table 5). For the comparison of the amount of seroma fluid between the groups, the seroma fluid volume in the non-closed group was greater than that in the closed group (34.48 ± 20.40 vs. 43.87 ± 16.40 ml, *P* = 0.006, 7 days) and (21.79 ± 8.42 vs. 30.74 ± 10.39 ml, *P* = 0.002, 14 days) after surgery. We found that there was no difference in the amount of seroma fluid between the two groups after 21 days (13.60 ± 5.17 vs. 14.69 ± 6.59 ml) and 28 days (11.75 ± 2.36 vs. 13.57 ± 1.99 ml) after surgery (*P* > 0.05) (Table 6)**.**

**Table 5:** Comparison of postoperative seroma cases between the two groups.

	Seroma cases (%)			
Group	7 days	14 days	21 days	28 days
Closed (*n* = 85)	45 (53%)	23 (27%)	10 (12%)	4 (5%)
Non-closed (*n* = 95)	79 (83%)	43 (45%)	29 (31%)	10 (11%)
*t*	19.112	6.402	9.304	2.119
*P*	<0.01	0.011	0.002	0.146

**Table 6:** Comparison of the postoperative amount of seroma fluid between the two groups.

	Amount of seroma fluid (ml)			
Group	7 days	14 days	21 days	28 days
Closed (*n* = 85)	34.48 ± 20.40	21.79 ± 8.42	13.60 ± 5.17	11.75 ± 2.36
Non-closed (*n* = 95)	43.87 ± 16.40	30.74 ± 10.39	14.69 ± 6.59	13.57 ± 1.99
*t*	2.777	3.303	0.474	1.371
*P*	0.006	0.002	0.638	0.860

The mortality of this series was 0%, but one patient had bulging of the abdominal wall in the inguinal region, which returned to normal after compression. No other seroma-related complications, such as hernia recurrence, infection, or mesh rejection, occurred during the study period.

## Discussion

Laparoscopic inguinal hernia repair has the advantages of a small incision, less bleeding, less pain, and faster recovery. Laparoscopic inguinal hernia repair has been increasingly affirmed. However, there is still controversy on the laparoscopic repair of Gilbert type III inguinal hernia or scrotal hernia (12). Due to the large defect of the hernia ring in indirect hernia, synthetic mesh bridging is often performed to repair it. The incidence of complications such as infection increases (13).

Laparoscopic internal ring suturing has been proven to be safe and effective for the treatment of inguinal hernia in adolescents (14). For small groins, the effect of a patch repair can also be achieved by suturing the internal ring, but for large hernias, the incidence of postoperative seroma swelling, pain, and equivalency is high. In addition, the literature reported that in the treatment of direct hernia under laparoscopy, the direct hernia cyst was eliminated by suturing the internal ring, which significantly reduced the incidence of postoperative seroma and did not increase the risk of postoperative infection, pain, or recurrence (15–17). A few reports on Gilbert type III indirect hernia and scrotal hernia stated that the incidence of seroma can also be reduced by closing the inner ring (11). For the cases of Gilbert type III indirect hernia and scrotal hernia prone to seroma and pain after surgery, it is assumed that the internal ring can also be closed to reduce complications.

Therefore, we randomly divided patients with Gilbert type III indirect hernia into a closed group and a non-closed group in a prospective study. Under the same basic conditions of the two groups, it was found that the operation time of the closed group with internal ring defect closure was longer than that of the non-closed group with unclosed hernia ring defects, which was related to the need for an inner ring closure during operation in the closed group. The suturing of the inner ring mainly involves injury to the vas deferens, genitofemoral nerve, spermatic cord, and inferior abdominal vessels. The suture location is in the upper part of the joint tendon and the lower iliopubic bundle. The suture depth should not be too deep in order to avoid injuring the nerves. A U-suture through the inferior epigastric vessels with a loose and tight compression of the spermatic cord led to scrotal swelling and even testicular ischemic necrosis, and inner ring suture relaxation did not affect the closed inner ring (11, 18). Therefore, the operation time of the experimental group was longer, but the average time was controlled within 15 min, which had no effect on the anesthetic effect of the patients. The choice of suture can include inverted thorn sutures and slow absorption sutures. Our center chooses slow absorption sutures because the cost is relatively economical and applicable, and there is basically no difference in the total operation cost between the two groups. After the TAAP/TEP operation, a normal diet was given 8 h after the operation, and the VAS score of pain on the first day after the operation was approximately 3. A few patients were given oral painkillers to relieve symptoms. Patients with type III hernia and scrotal hernia usually have a long history, especially elderly individuals, with more basic diseases and more urinary retention incidence after operation. At the same time, a few patients have other complications, such as intestinal obstruction, nausea, vomiting, and card incision bleeding. All of these complications had resolved and returned to normal after symptomatic treatment and observation. Therefore, there was no significant difference in postoperative hospital stay or the incidence of short-term postoperative complications between the two groups, and the patients were satisfied with the outcome after the operation.

Although laparoscopic surgery produces less postoperative pain than open surgery, postoperative pain in the operative area is a common complication after laparoscopic inguinal hernia repair with an incidence of approximately 2%–20% (18, 19), which is one of the main reasons affecting the quality of life of patients who underwent surgery. We found no significant difference in the pain incidence and VAS score between the two groups on the first postoperative day, while with the prolongation of postoperative time, the pain incidence and the VAS score in both groups showed a continuous trend of reduction after surgery, reflecting the physiological changes secondary to the patients' gradual recovery and pain reduction after surgery. This process is one of the advantages of laparoscopic inguinal hernia repair. However, the incidence of pain was significantly higher in the control group than in the experimental group at 7, 14, and 21 days after surgery, and the VAS scores of postoperative pain were also found to be higher in the control group than in the experimental group at 7 and 14 days after surgery. In our practice of inner ring closure, the structures of blood vessels and spinal cord are constant and obvious and can be easily avoided. Although the nerves are not easy to see and their positions are different, these two groups of nerves [the GFN and lateral femoral cutaneous nerve (LFCN)] pass under the iliac pubic tract and through the transverse abdominal muscle to reach the inguinal box. The suture is only placed between the iliac pubic tract and the joint tendon. The direction of the suture is basically parallel to the nerve, and the suture needle is visible to avoid reaching too deep. This also avoids the risk of pain caused by suturing the inner loop (11, 19, 20). There are various causes of postoperative pain. In addition to the pain that may result from surgical procedures, changes in the volume of seroma effusion after surgery can also cause varying degrees of pain (21).

The incidence of seroma after inguinal hernia surgery has been shown to be one of the most significant complications, ranging from 0.5% to 12.2% after TEP, 3.0% to 8.0% after TAPP, and up to 80% in type III and scrotal hernias (22). The factors influencing the development of seroma are multifaceted, and patients with a long medical history or large hernia sacs are at high risk for seroma development. Also, this also occurs in patients with underlying diseases such as diabetes mellitus, hypertension, or low protein levels in the body, which reduce the ability of tissue regeneration and the efficiency of absorption of inflammatory substances. The main mechanism for the development of seroma is the inclusion of the peritoneum of the wall of the hernia sac stump, obstruction of lymphatic flow and reduced absorption of the open hernia sac, inflammatory exudation secondary to the trauma of herniorrhaphy, and the continuous secretion of fluid after surgery due to the foreign body stimulation of the surgical wound by the patch, as well as the low position of the open hernia sac, so that the fluid accumulates and is difficult to absorb in the short term, forming a seroma (5, 23, 24).

The occurrence of postoperative seroma affects the outcome of surgical repair and leads to postoperative pain, infection, and recurrence. Most inguinal hernias of types I and II are self-absorbing after surgery. However, type III and scrotal hernias have large postoperative hernia sac stump and a high incidence of seroma, and one problem to be solved is the reduction in the incidence of seroma and the amount of fluid accumulation (25). In the available reports, whether the hernia sac is transected or not has not been shown to be effective in reducing the incidence of seroma and the amount of fluid accumulation, which can be prevented in the short term by giving prophylactic placement of a drainage tube in the trabecular cavity, but after removal of the drainage tube, the seroma reappears. Therefore, at present, the only way to reduce and prevent seroma is to aspirate the effusion to treat the symptoms at the time, and there is no real effective way to reduce and prevent it (8, 26, 27).

Closing the hernia ring in direct hernia repair has proven to be effective in preventing postoperative seroma. By closing the inner ring, we found that on the 7th, 14th, and 21st days after surgery, the incidence of seroma cases was significantly lower than that of the non-closed group, and on the 7th and 14th days after surgery, the cumulative amount of seroma fluid in the non-closed group was also significantly higher than that in the closed group.

Interestingly, through experimental data, we found a correlation between the incidence of postoperative pain, VAS scores, and the incidence of seroma tumors in the two groups of patients. The seroma and pain incidence rates in the non-closed group were significantly higher than those in the closed group with internal ring closure at 7, 14, and 21 days postoperatively, and the VAS scores and seroma volume in the non-closed group were simultaneously higher than those in the closed group at 7 and 14 days postoperatively. With the extension of the postoperative time, the postoperative pain and seroma in both groups gradually decreased without differences. Therefore, we can preliminarily conclude that there is a significant relationship between postoperative pain and seroma for large indirect hernia or scrotal hernia repair, and there is a positive correlation between postoperative pain, seroma, and fluid accumulation. The seroma in the stump space cannot be absorbed automatically within a short period of time and gradually accumulates, leading to pain and discomfort in the inguinal region. When seroma tumors occur and the fluid volume decreases, the postoperative pain is significantly alleviated and disappears.

## Conclusion

Laparoscopic closure of the internal ring in Gilbert type III inguinal hernia is safe and effective, especially in reducing the occurrence of postoperative seroma and alleviating postoperative pain. In addition, after closure of the internal annulus, as in strengthening the posterior wall of the inguinal box, a lightweight large mesh patch can be placed, even without fixation, to further reduce postoperative pain and other postoperative complications and to improve the experience of care without increasing the cost of patient care.

## Data Availability

The original contributions presented in the study are included in the article; further inquiries can be directed to the corresponding authors.
